# Weed detection and recognition in complex wheat fields based on an improved YOLOv7

**DOI:** 10.3389/fpls.2024.1372237

**Published:** 2024-06-24

**Authors:** Kaixin Wang, Xihong Hu, Huiwen Zheng, Maoyang Lan, Changjiang Liu, Yihui Liu, Lei Zhong, Hai Li, Suiyan Tan

**Affiliations:** College of Electronic Engineering, South China Agricultural University, Guangzhou, China

**Keywords:** wheat fields, wheat weed detection, CARAFE, SE, CoT, WIoU

## Abstract

**Introduction:**

The precise detection of weeds in the field is the premise of implementing weed management. However, the similar color, morphology, and occlusion between wheat and weeds pose a challenge to the detection of weeds. In this study, a CSCW-YOLOv7 based on an improved YOLOv7 architecture was proposed to identify five types of weeds in complex wheat fields.

**Methods:**

First, a dataset was constructed for five weeds that are commonly found, namely, *Descurainia sophia*, thistle, golden saxifrage, shepherd’s purse herb, and *Artemisia argyi*. Second, a wheat weed detection model called CSCW-YOLOv7 was proposed to achieve the accurate identification and classification of wheat weeds. In the CSCW-YOLOv7, the CARAFE operator was introduced as an up-sampling algorithm to improve the recognition of small targets. Then, the Squeeze-and-Excitation (SE) network was added to the Extended Latent Attention Networks (ELAN) module in the backbone network and the concatenation layer in the feature fusion module to enhance important weed features and suppress irrelevant features. In addition, the contextual transformer (CoT) module, a transformer-based architectural design, was used to capture global information and enhance self-attention by mining contextual information between neighboring keys. Finally, the Wise Intersection over Union (WIoU) loss function introducing a dynamic nonmonotonic focusing mechanism was employed to better predict the bounding boxes of the occluded weed.

**Results and discussion:**

The ablation experiment results showed that the CSCW-YOLOv7 achieved the best performance among the other models. The accuracy, recall, and mean average precision (mAP) values of the CSCW-YOLOv7 were 97.7%, 98%, and 94.4%, respectively. Compared with the baseline YOLOv7, the improved CSCW-YOLOv7 obtained precision, recall, and mAP increases of 1.8%, 1%, and 2.1%, respectively. Meanwhile, the parameters were compressed by 10.7% with a 3.8-MB reduction, resulting in a 10% decrease in floating-point operations per second (FLOPs). The Gradient-weighted Class Activation Mapping (Grad-CAM) visualization method suggested that the CSCW-YOLOv7 can learn a more representative set of features that can help better locate the weeds of different scales in complex field environments. In addition, the performance of the CSCW-YOLOv7 was compared to the widely used deep learning models, and results indicated that the CSCW-YOLOv7 exhibits a better ability to distinguish the overlapped weeds and small-scale weeds. The overall results suggest that the CSCW-YOLOv7 is a promising tool for the detection of weeds and has great potential for field applications.

## 1 Introduction

Wheat is a commonly cultivated cereal worldwide that covers approximately 237 million hectares annually, producing 765 million tons of yield. However, weeds are threatening wheat yield by competing with crops for resources (e.g., water, light, and nutrients) and providing hosts for diseases and pests (Ying et al., 2021). Up to 40% of global crop production is lost each year due to weeds, pests, and diseases (Tang et al., 2023). Thus, it is important to adopt weed management to reduce yield losses. Traditional weed control strategies, such as mechanical and chemical approaches, are known to be time-consuming, labor-intensive, and potentially harmful to the surrounding environment (Scavo and Mauromicale, 2021; Sharma et al., 2021; Monteiro and Santos, 2022). Site-specific weed management (SSWM) is an essential approach that helps to counteract the issue of the overuse of herbicides. The precise detection of weeds in the field is the premise of implementing SSWM. However, the similar color, shape, and occlusion between wheat and field weeds pose a challenge to the detection of weeds in wheat fields.

The development of machine vision and image processing technologies has enabled the application of more accurate and efficient weed identification techniques. A variety of sensor technologies, including RGB, multispectral, and hyperspectral sensors, have been utilized to capture detailed image features of crops and weeds. These images are then analyzed using different segmentation, feature extraction, and classification techniques, allowing for precise identification of weeds and mapping of weed distribution with high accuracy. Sulaiman et al. (2022) presented the application of hyperspectral remote sensing imagery (HRSI) for the detection of weeds, listing common weed species and their reflectance in specific bands and using the algorithms and models in the analysis of weed discrimination. Xia et al. (2022) utilized unmanned aerial vehicles (UAVs) to acquire multispectral and RGB images. Image fusion technology was employed to augment available information, and a weed spectral resistance index [WSRI = (RE − R)/(RE − B)] was developed based on the disparity between susceptible and resistant weed biotypes. Furthermore, a deep convolutional neural network (DCNN) was deployed to evaluate the viability of identifying resistant weeds in the field. Parra et al. (2020) presented the use of edge-detection techniques to identify weed presence. Twelve edge-detection filters were tested, using aggregation techniques applied to three filters to reduce false positives. The performance in ornamental was 80% and 83% in terms of Pre and F1, respectively.

In terms of algorithms, machine learning technology has been widely used in recent years to meet the growing demand for fast, accurate, and non-destructive applications in weed identification. In the traditional machine learning algorithms, texture, color, shape, and thermal features, extracted from different sensor images, are used alone or in combination and then adopted in the machine learning algorithms to finish weed detection. Su et al. (2022) proposed an integrated approach that combines UAV technology, multispectral imagery, and machine learning techniques. Random Forest classifier with Bayesian hyperparameter optimization was used as the classification algorithm to enhance model simplicity and empirical interpretability. Sohail et al. (2021) utilized texture and color features extracted from images and used the Random Forest algorithm to train a model using extracted feature descriptors. The performance of the model was evaluated based on regression metrics, precision, recall, and F1 scores. The results demonstrated that the model achieved a high accuracy rate of 91% for weed classification. Zamani and Baleghi (2023) collected 100 pairs of visible and thermal images of rice and weeds. Through image segmentation, feature vectors containing 15 morphological, 12 spectral, 10 textural, and 11 new thermal features were extracted. To optimize feature selection, genetic algorithms (GAs) were employed. Multiple late and early fusion structures were developed at the decision level. Zheng et al. (2017) developed a corn detection method based on color features using a post-processing algorithm to differentiate between corn and weeds. Feature selection using principal component analysis aimed to reduce the effect of light, and finally support vector was used as a classifier. The results showed that the color index used performed consistently under different weather and time of day.

Unlike traditional machine learning, deep learning algorithms are a new era of machine learning, and the step of feature extraction is performed by the deep learning models themselves. Deep learning algorithms have already achieved better results in object detection than traditional machine learning algorithms. Zou et al. (2022) proposed a modified U-net for segmenting wheat and weeds in images and used an image classification task to select the backbone network for the encoding part. The results showed that the Intersection over Union (IoU) of segmentation reached 88.98%. Guo et al. (2023) proposed WeedNet-R to extend the sensory field of the entire network by adding numerous context modules to RetinaNet’s neck. The mean accuracy [mean average precision (*mAP*)] of weed detection in sugar beet fields was improved by 4.65% to 92.30%. Jiang et al. (2020) proposed a graph convolutional network (GCN) approach based on a CNN feature. A GCN graph was constructed from the extracted weed CNN features and their Euclidean distances and enriched the model by exploiting labeled and unlabeled image features. The approach satisfied the real-time requirement of the field of weed control. Kim and Park (2022) proposed a multi-task semantic segmentation-convolutional neural network for detecting crops and weeds (MTS-CNN) using one-stage training. This approach heightened the correlations between crop and weed classes by adding the crop, weed, and both (crop and weed) losses. Xu et al. (2021) proposed a framework based on multi-modal information fusion for accurate detection of weeds in wheat fields in a natural environment, overcoming the limitation of single modality in weed detection. Wang Y. et al. (2023) proposed a fine-grained weed recognition method based on Swin Transformer and two-stage transfer learning, which can improve the recognition performance of weeds and crops with similar visual characteristics. The results showed that the proposed method achieved a precision of 99.33%. The YOLO series is a typical regression-based target detection algorithm, which has now evolved to YOLOv8. Wang A. et al. (2022) proposed a pixel-level synthesization data augmentation method and a TIA-YOLOv5m network. This model added a transformer encoder block to the backbone, used a channel feature fusion with involution (CFFI) strategy, and introduced adaptive spatial feature fusion (ASFF) for feature fusion of different scales in the prediction head. The results showed that mAP0.5was 90.0%. Zhang et al. (2022) proposed an EM-YOLOv4-Tiny network incorporating multiscale detection and attention mechanisms. An Efficient Channel Attention (ECA) module was added to the Feature Pyramid Network (FPN) of YOLOv4-Tiny, and the soft Non-Maximum Suppression (soft-NMS) was adopted. Although the network improved the recognition accuracy of the model compared with the original YOLOv4-Tiny network, it also increased the volume of the model to a certain extent. Wang H. et al. (2023) used the YOLOv6 algorithm to identify surface defects in the lock body workpiece, and an improved algorithm based on the Canny-Devernay was also used for sub-pixel edge detection to determine the size of the bead hole of the lock cylinder; the results showed that the average accuracy was 0.911 and the average inaccuracy was less than 0.03 mm. Wang C. et al. (2023) combined the YOLOv8 model with monocular and binocular image processing techniques for the identification and localization of lychee picking points and developed intelligent control algorithms to actively remove obstacles in conjunction with the obstacle situation at the picking point. The results showed that the developed lychee-picking robot can effectively realize obstacle removal.

Based on the above research, deep learning algorithms have become the mainstream weed detection methods and have shown promising performance. The one-stage network, YOLO, has the advantages of high accuracy and speed and has been improved in different ways to achieve better performance. However, challenges, such as similar morphology of wheat and weeds, multi-scale weeds, and occluded plants, still block the way in improving detection accuracy in complex field environments. Therefore, an improved YOLOv7, namely, the CSCW-YOLOv7, was proposed to identify five types of weeds in complex wheat fields. The contributions of this study are as follows. First, the dataset of five weeds, namely, *Descurainia sophia*, thistle, golden saxifrage, shepherd’s purse herb, and *Artemisia argyi*, that are commonly found in wheat fields were constructed. Second, based on an improved YOLOv7, a wheat weed detection model called the CSCW-YOLOv7 was proposed to achieve the accurate identification and classification of wheat weeds. In the CSCW-YOLOv7, the CARAFE operator was introduced into the YOLOv7 network as an up-sampling algorithm to improve the recognition of small targets. Then, the SE was added to the Extended Latent Attention Networks (ELAN) module in the backbone network and the concatenation layer in the feature fusion module to enhance important weed features and suppress irrelevant features. In addition, the contextual transformer (CoT) module, a transformer-based architectural design, was used to capture global information and enhance self-attention by mining contextual information between neighboring keys. Finally, the Wise Intersection over Union (WIoU) loss function, which introduces a dynamic non-monotonic focusing mechanism, was employed to better predict the bounding boxes of the occluded weed. After the CSCW-YOLOv7 construction, the performance of the model was comprehensively evaluated.

## 2 Materials and methods

### 2.1 Image acquisition and preprocessing

#### 2.1.1 Image acquisition

**Figure 1:**
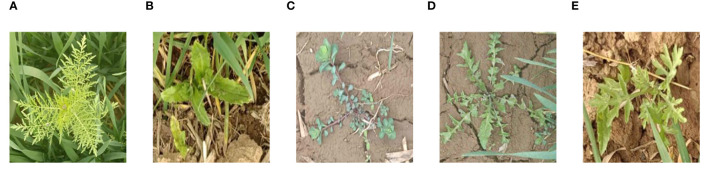
Sample of wheat weed data: **(A)** *Descurainia sophia*, **(B)** thistle, **(C)** golden saxifrage, **(D)** shepherd’s purse herb, and **(E)** *Artemisia argyi*.

In this study, wheat and its common accompanying weeds in the natural environment were used as the experimental subjects, and the image collection site was located in a wheat planting farm near the Quma line in Potou Town, Jiyuan City, Henan Province, PR China (112°27′37″E, 34°57′52″N). The best weeding period of the wheat field is the regreening stage; therefore, the regreening stage was selected for weed data collection. Weed images were collected from March 10 to 25, 2023. The collected images included five kinds of weeds that are commonly found in wheat fields, namely, *D. sophia*, thistle, golden saxifrage, shepherd’s purse herb, and *A. argyi*; their scientific names are *D. sophia*, *Cirsium arvense* var. *integrifolium*, *Euphorbia esula* L., *Capsella bursa-pastoris*, and *A. argyi*, respectively. A sample of each kind of weed in the dataset is given in **Figure 1**. Smartphone ViVO Y52s with an image resolution of 4,000 pixels × 3,000 pixels was adopted to capture the weed images. To include diverse weed samples and construct a comprehensive weed dataset, weed images were collected in the natural field environment and under different conditions, and a total of 2,614 original images were collected. First, weed images were collected at different times of the day: 8:30–10:30, 13:30–15:30, and 17:00–18:00. Second, images were collected at different weather conditions, including sunny and cloudy days. Furthermore, the shooting angle was vertically downward with the height varying from 30 to 60 cm from the ground.

**Figure 2:**
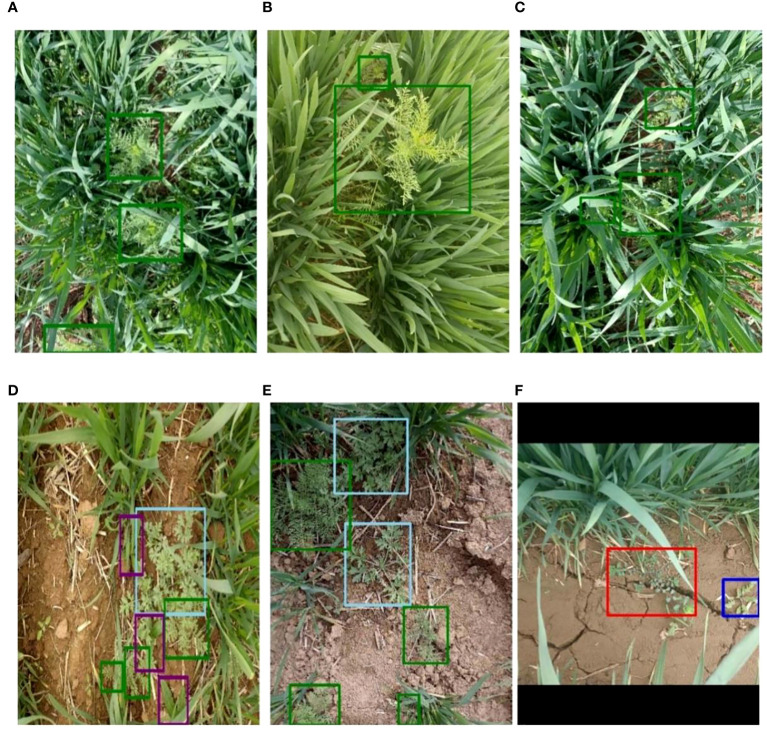
Example images of weeds in wheat fields. **(A)** Light and shade. **(B)** Weeds of different sizes. **(C)** Mutual occlusion between wheat and weeds. **(D)** Similar morphology of wheat and weeds. **(E, F)** Appearance of different weed species. different colors of the bounding boxes in the images represent different weed species.

**Figure 3:**
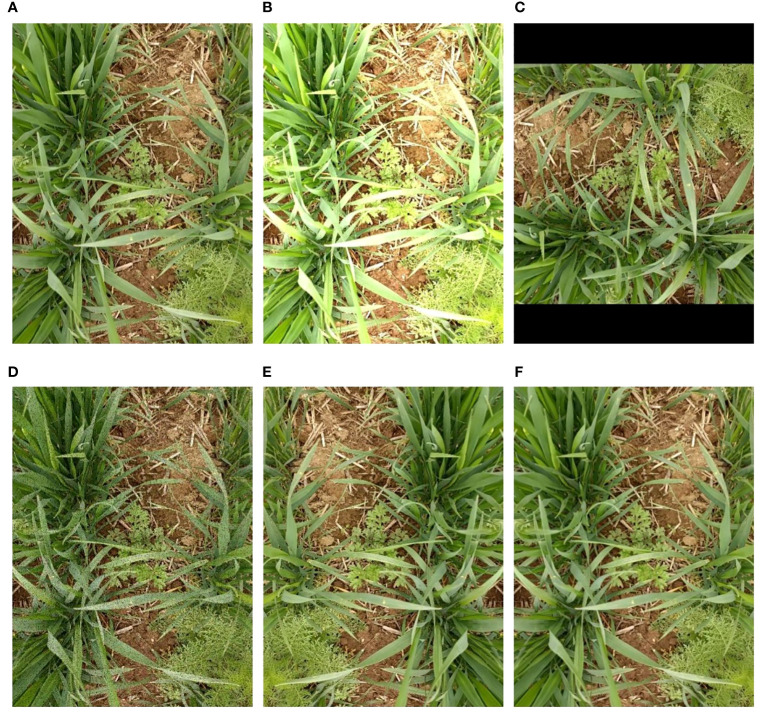
Illustration of five image augmentations on weed images: **(A)** origin, **(B)** brightness adjustment, **(C)** rotation, **(D)** noise addition, **(E)** image flipping, and **(F)** image blur. different colors of the bounding boxes in the images represent different weed species.

**Figure 4:**
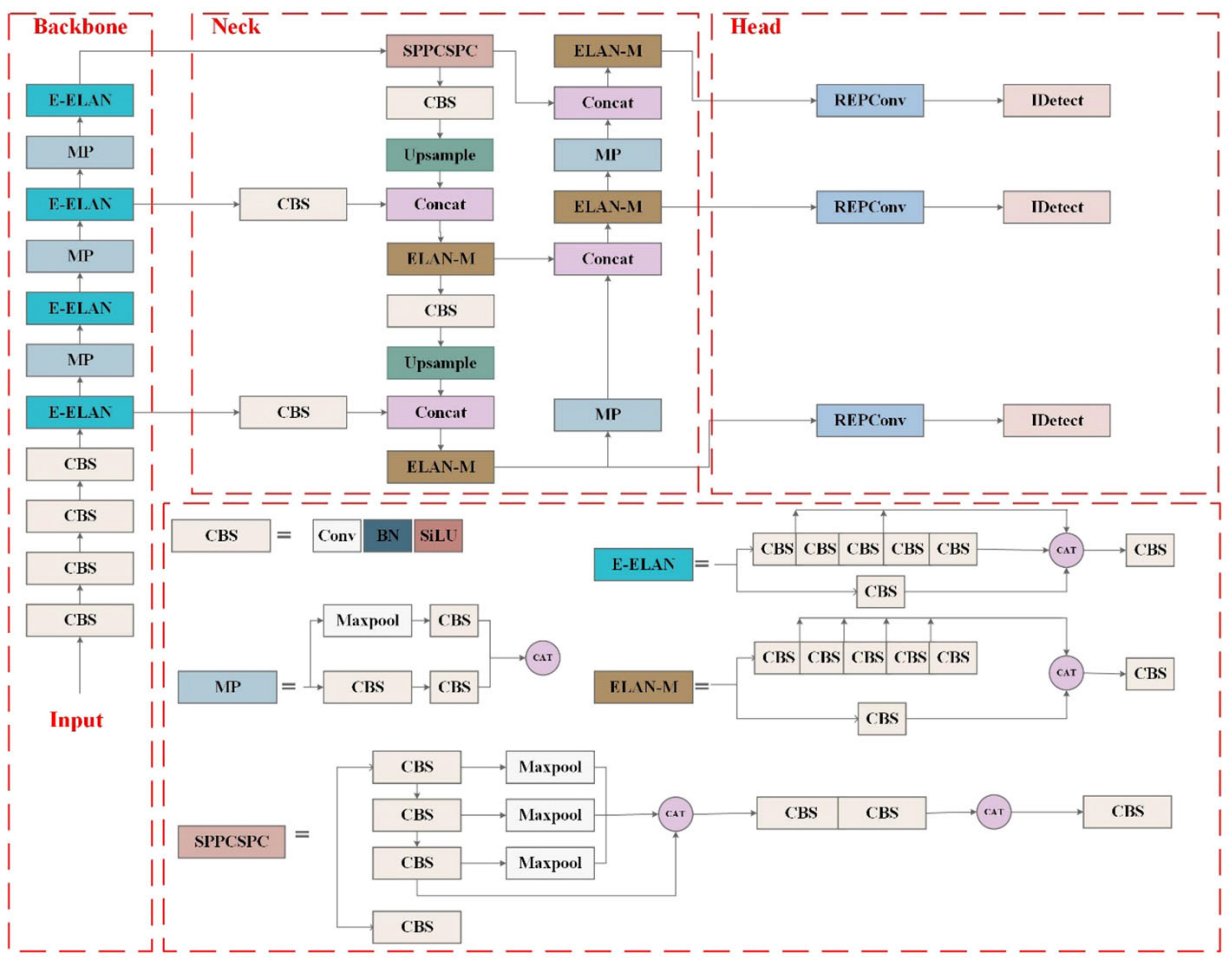
YOLOv7 network structure diagram.

Therefore, the constituted weed dataset brings great challenges to the method of weed detection because of the diversity and complexity of the phenotyping of weeds: 1) complex field background, including water reflection, shade, and light (**Figure 2A**); 2) great variation of weeds in size, color, and shape caused by light conditions, varieties, and image shooting angles (**Figure 2B**); 3) mutual occlusion between wheat and weeds (**Figure 2C**); 4) similar morphology of wheat and weeds (**Figure 2D**); and 5) appearance of different weed species (**Figures 2E, F**).

#### 2.1.2 Image preprocessing and dataset preparation

After image acquisition, the annotation tool LabelImg was used for manual annotation to obtain the ground truth of weeds for subsequent training. Weeds were labeled using bounding boxes following the standard format of YOLO. Bounding boxes were minimum external rectangles that contained all pixels of the weeds. In case of weeds of irregular shape or patches of weeds, multiple bounding boxes were drawn to include the entire weed features. In addition, different colors of the bounding boxes were used to draw and recognize the category of weed class. In our study, green, purple, red, dark blue, and light blue boxes were used to label weeds of *D. sophia*, thistle, golden saxifrage, shepherd’s purse herb, and *A. argyi*, respectively. **Figure 2** shows image annotations of five kinds of weeds. Specifically, *D. sophia*, thistle, golden saxifrage, shepherd’s purse herb, and *A. argyi* were labeled with 11,136, 11,234, 882, 1,675, and 1,884 annotation bounding boxes in the original images, respectively. After labeling, a txt file was automatically generated containing the category and coordinate information for each labeled box in each image, recorded as the label id, the coordinates of the center point of the labeled box (x, y), and the width and height of the labeled box (w, h) in order to determine the relative position of the weed target in the image.

Furthermore, data augmentation, a method of artificially enlarging datasets, was conducted on the original images to enhance the generalization ability of the weed detection models and prevent overfitting. Five traditional image augmentation techniques including brightness adjustment, rotation, image flipping, noise addition, and image blur were adopted. Meanwhile, the bounding box information was preserved during image augmentation. Examples of image augmentation are shown in **Figure 3**. Due to the imbalance distribution of weed species, data augmentation with different magnifications was used for different weed species to reduce the imbalance. After image augmentation, the number of *D. sophia* and thistle was 2.6 times and 4.3 times larger than the original images, respectively. Golden saxifrage, shepherd’s purse herb, and *A. argyi* were 16 times, 10.8 times, and 13.4 times larger than the original images, respectively. Finally, the augmented datasets were randomly divided into training sets, validation sets, and test sets. The validation sets were part of the training sets. Specifically, 80% were training sets, 20% of which were validation sets, and the remaining 20% were test sets. The training sets were used to train the model and determine its parameters, while the validation sets were used to determine the network structure and adjust the parameters of the model. The test sets aim to test the generalization ability of the model. The detailed information on the datasets is shown in **Table 1**.

**Table 1:** Distribution table of wheat weed dataset.

Weeds	Origin images	Number of annotation boxes	Image after augmentation	Number of training dataset	Number of annotation boxes in training dataset	Number of test dataset	Number of annotation boxes in test dataset
*Descurainia sophia*	1,841	11,136	4,811	3,739	8,675	1,072	2,461
Thistle	639	11,234	2,753	2,134	8,605	619	2,629
Golden saxifrage	19	882	304	236	696	68	186
Shepherd’s purse herb	62	1,675	668	519	1,287	149	388
*Artemisia argyi*	53	1,884	710	555	1,468	155	416

### 2.2 Construction of wheat weed detection model based on an improved YOLOv7

#### 2.2.1 YOLOv7

YOLOv7, proposed by the original YOLOv4 research team in July 2022 (Bochkovskiy et al., 2020), is one of the most advanced one-stage object detection algorithms that balance the conflict between the number of parameters, computational consumption, and performance, achieving satisfactory results in terms of speed and accuracy (Wang C.-Y. et al., 2022). The significant improvements of YOLOv7 lie in four aspects, including efficient ELAN module, re-parametrization modules, label assignment strategies, and auxiliary head training strategy. The main structure of YOLOv7 consists of four components, Input, Backbone, Neck, and Head, as shown in **Figure 4**. In the Input, images after preprocessing and enhancement, including mixup and mosaic, are rescaled to 640 pixels and then fed into the Backbone. The Backbone, responsible for feature extraction, is composed of 51 layers (Layer0–50) and mainly includes modules of standard CBS (Conv-BN-SiLU), ELAN, and max pooling layers (MP). The CBS employs three different convolutional kernel sizes and step sizes to generate features at various scales. The ELAN module continuously enhances the network’s learning ability by controlling the shortest and longest gradient path. Simultaneously, it encourages the network to learn more diverse and discriminative features by enhancing the interaction between each feature layer through expansion, random combination, and splicing. The MP module integrates two down-sampling branches with pooling and convolution, and it utilizes max-pooling operations to reduce the spatial dimension of the feature maps. At last, the Backbone layer outputs feature information of different sizes, which are located in the 24th, 37th, and 50th layers. The Neck is designed to perform feature extraction and fusion. First, the Spatial Pyramid Pooling, Cross Stage Partial Channel (SPPCSPC) network employs four max pooling layers of different kernel sizes to obtain different receptive fields, thereby helping to better extract features of different scales. Then, feature fusion is performed by up-sampling and down-sampling features of different scales obtained from the backbone, following the PANet structure in the YOLOv5m network. The Neck outputs feature maps of different sizes in the 75th, 88th, and 101st layers. The reparameterized RepConv network structure is introduced to the Head for training and achieves recognition and classification of images (Ding et al., 2021). Moreover, the Head module for the first time adds an auxiliary head for loss calculation in the middle of the network to assist training, namely, the auxiliary head training strategy, improving the performance by multi-way branching during the training process.

#### 2.2.2 CSCW-YOLOv7 construction based on an improved YOLOv7

**Figure 5:**
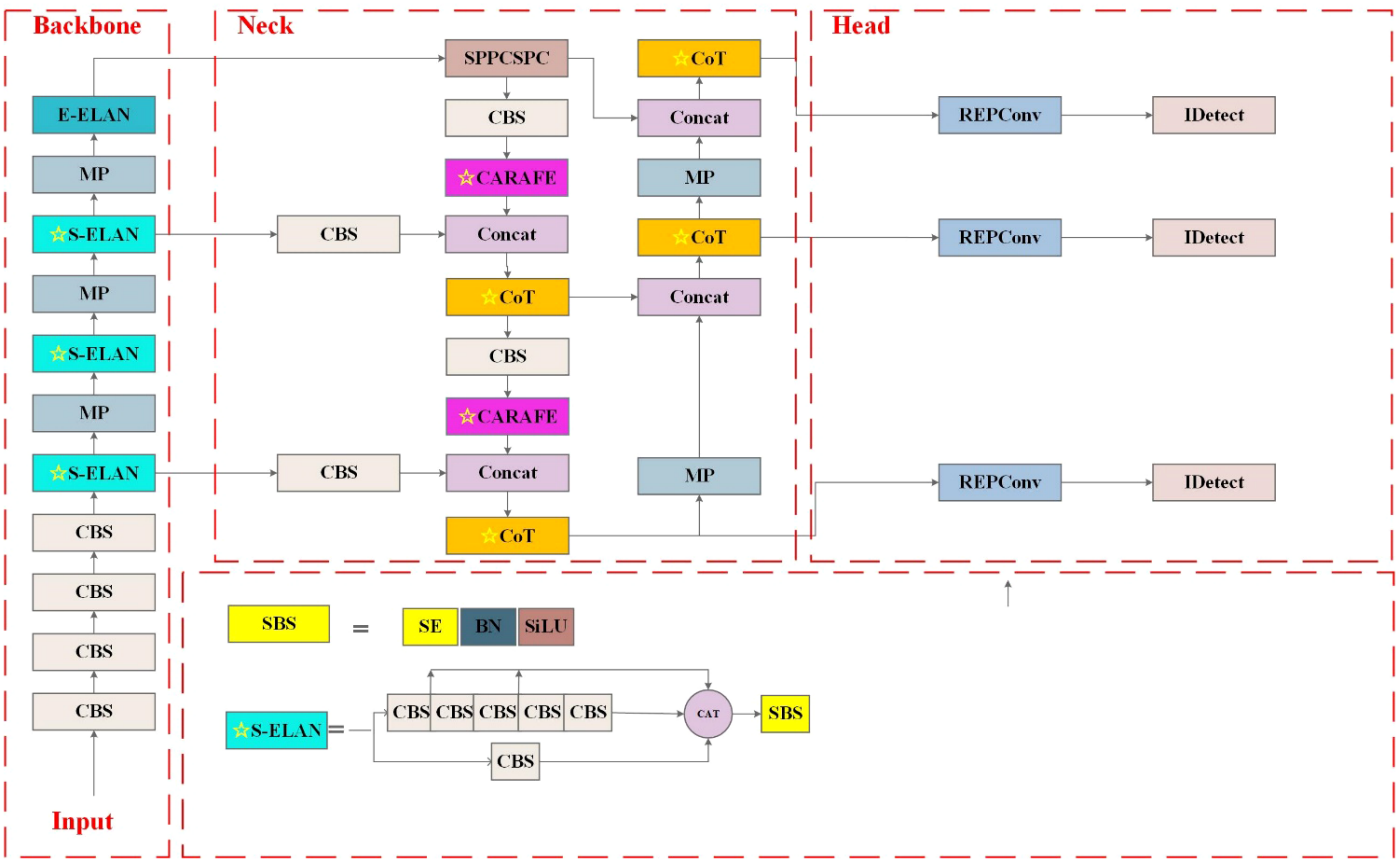
Improved YOLOv7 network structure diagram. The ☆ indicates for modifications.

In practice, the in-field wheat weed recognitions are still facing many challenges: 1) complex field background, including water reflection, shade, and light; 2) great variation of weeds in size, color, and shape caused by light conditions, varieties, and image shooting angles; 3) mutual occlusion between wheat and weeds; and 4) similar morphology of wheat and weeds. These challenges motivate the development of wheat weed detection algorithms that can operate over images taken under a variety of conditions. This study adopts YOLOv7 as the baseline model and investigates further optimizations to enhance its performance on wheat weed detection and classification. The proposed CSCW-YOLOv7 model is shown in **Figure 5**. First, the CAREFE up-sampling method is adopted to improve the recognition of small targets. Second, to enhance important weed features and suppress irrelevant features, the SE attention mechanism is used. Then, the ELAN-M module is replaced with the CoT module to capture global information and enhance self-attention by mining contextual information between neighboring keys. Finally, the WIoU loss function is employed to improve the accuracy of the detection results and the convergence speed of the network.

#### 2.2.3 CARAFE operator

YOLOv7 adopts the nearest-neighbor interpolation up-sampling method, which shows the advantages of simplicity and low computational cost. However, nearest-neighbor interpolation only considers adjacent pixels and does not fully utilize the semantic information of the feature map, resulting in discontinuous grayscale values after resampling and loss of image quality. To obtain a larger receptive field and better detect the small wheat weed, the CARAFE (Xu et al., 2021) operator is employed in the up-sampling method in this paper, which is proven to have the advantages of having a large receptive field and being content-aware and lightweight. The CARAFE can dynamically generate an adaptive kernel based on the input feature map without introducing too many parameters and calculations, thus making better use of the surrounding pixel information while maintaining lightweight.

**Figure 6:**
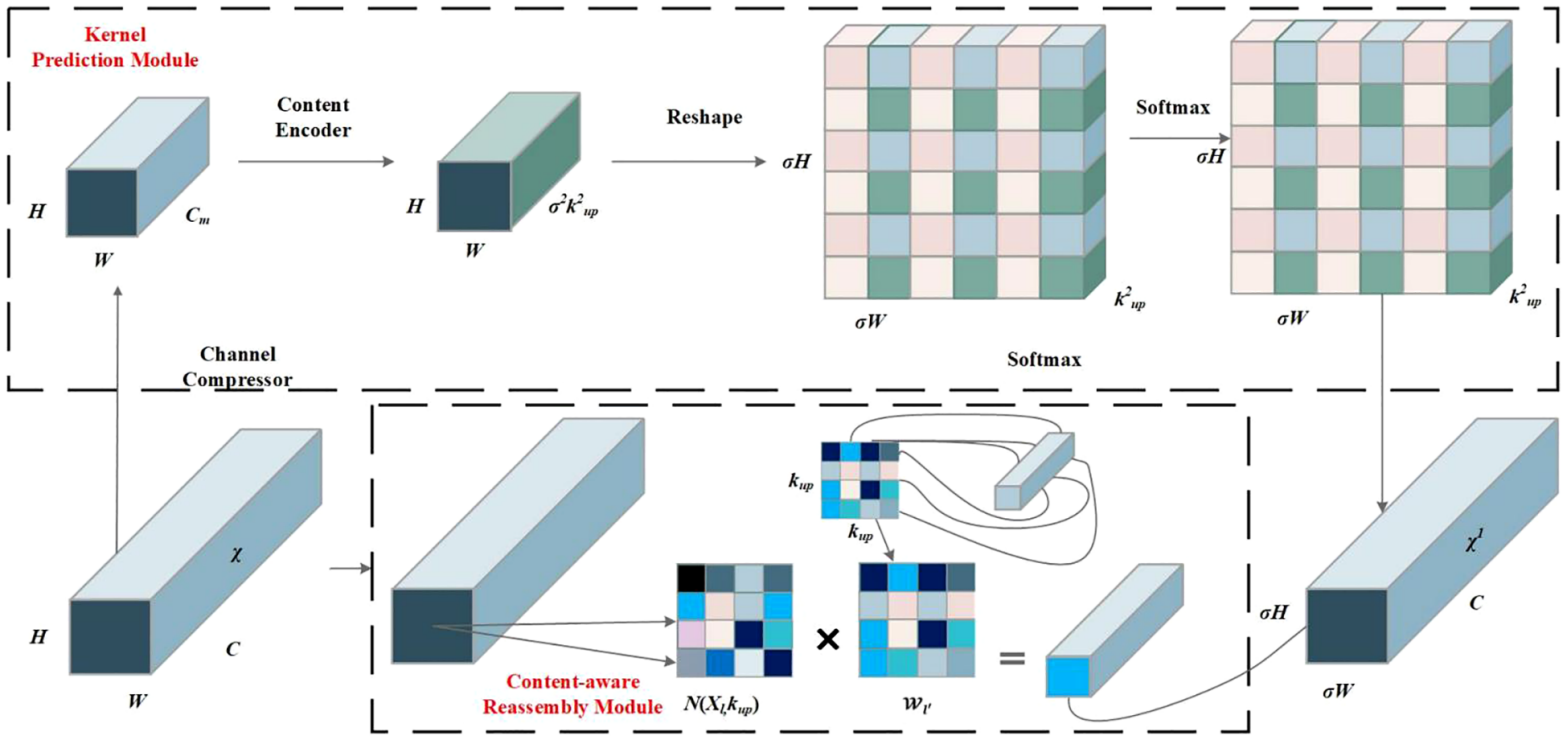
CARAFE operator structure diagram.

CARAFE consists of two modules: a kernel prediction module and a content-aware reassembly module. The CARAFE structure is shown in **Figure 6**. The kernel prediction module falls into three steps: channel compression, content encoding, and kernel normalization. First, the feature map with the shape of *H* × *W* × *C* is compressed to *H* × *W* × *C_m_* using a 1 × 1 convolution, where *C_m_* is the number of compressed channels. The channel compression reduces the amount of computation in subsequent operations. Then, a convolutional layer of kernel size *K_encoder_* × *K_encoder_* is utilized to generate reassembly kernels based on the compressing feature. Assuming that the number of input channels is *C_m_* and the number of output channels is *σ* ^2^ *k* ^2^ *_up_*, the channel dimension is expanded in the spatial dimension to obtain an up-sampling kernel with the shape of *σH* × *σW* × *k_up_* × *k_up_*. Finally, the softmax function is employed to normalize the predicted up-sampling kernels and make the convolutional kernel weights add up to 1. In the content-aware reassembly module, each position in the output feature map is mapped back to the input feature map, and a feature map of size *σH* × *σW* × *C* is obtained by taking a region of size of *k_up_* × *k_up_* centered on it. A dot product operation is performed with the up-sampling kernel of the predicted point. Different channels in the same position share the same up-sampling kernel.

#### 2.2.4 SE attention mechanism

**Figure 7:**
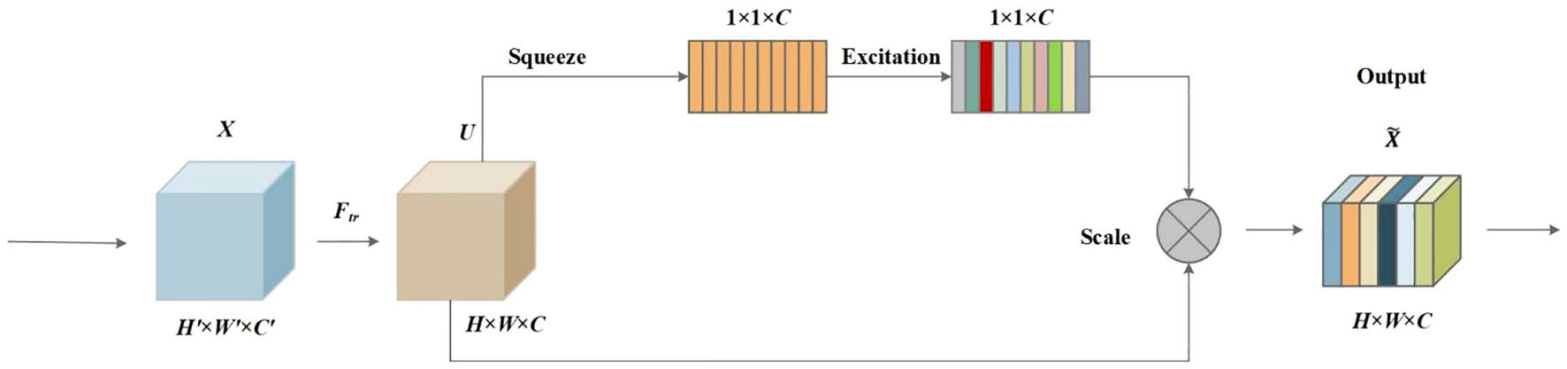
SE attention mechanism.

To better extract the target information and suppress the background information, the attention mechanism technique has been widely used in deep learning models. The SE attention network, proposed by Hu et al. (2017), is an architectural unit composed of squeeze and excitation blocks to use global information to selectively emphasize informative features and suppress less useful ones, and it follows three steps including the squeeze operation, excitation operation, and rescaling operation. The SE attention network was added to the ELAN module in the backbone network in this paper. The structure of the SE network is depicted in **Figure 7**.

SE block is built upon a transformation $F_{tr}$ mapping an input $X\in R_{}^{H^{\prime }\times W^{\prime }\times C^{\prime }}$ to feature maps $U\in R^{H\times W\times C}\text{. }F_{tr}$ is a convolutional operator, and $V=\left[v_{1},v_{2},\ldots ,v_{C}\right]$ was used to denote the learned set of filter kernels, where $v_{C}$ refers to the parameters of the *c*th filter. Then, the outputs were written as $U=\left[u_{1},u_{2},\ldots ,u_{C}\right]$ , where Equation 1:


(1) $$u_{c}=v_{c}\;*\;X=\sum_{\text{s}=1}^{C'}v_{c}^{s}\;*\;x^{s}$$

Then, the squeeze operation was followed. The global spatial information was squeezed into a channel descriptor using global average pooling. Therefore, a feature map of size *H* × *W* × *C* was compressed into a size of 1 × 1 × *C*. Formally, a statistic $z\in R^{c}$ is generated by shrinking *U* through its spatial dimensions *H* × *W* such that the *c*th element of z is calculated by Equation 2:


(2) $$z_{c}=F_{sq}\left(u_{c}\right)=\frac{1}{H\times W}\sum_{i=1}^{H}\sum_{j=1}^{W}u_{c}\left(i,j\right)$$

Furthermore, a simple gating mechanism with a sigmoid activation was employed to make use of the information aggregated in the squeeze operation and fully capture channel-wise dependencies. The gating mechanism follows the following Equation 3:


(3) $$s=F_{ex}\left(z,W\right)=\sigma \left(g\left(z,W\right)\right)=\sigma \left(W_{2}\delta \left(W_{1}z\right)\right)$$

where δ refers to the ReLU function, $W_{1}\in R^{\frac{C}{r}\times C}$ and $W_{2}\in R^{C\times \frac{C}{r}}$ .

At last, the scale operation of the channel-wise multiplication between the scalar $s_{c}$ and the feature map $u_{c}\in R^{H\times W}$ was followed by the Equation 4:


(4) $$\begin{array}{c} F_{\text{scale}}\left(s_{c},u_{c}\right)=\tilde{x_{c}}=s_{c}\times u_{c} \end{array}$$

where $\tilde{X}=\left[\tilde{x_{1}},\tilde{x_{2}},\ldots ,\tilde{x_{c}}\right]$ .

#### 2.2.5 Contextual transformer networks

In this work, a new transformer-style architecture, named CoT block, was employed and replaced with standard convolutions in the Neck of the YOLOv7 to exploit the contextual information among input keys and facilitate self-attention learning. Unlike the conventional self-attention mechanism of the transformer, the CoT block, designed by Li et al. (2021), combines context mining among neighbor keys and self-attention learning over a feature map with a favorable parameter budge.

**Figure 8:**
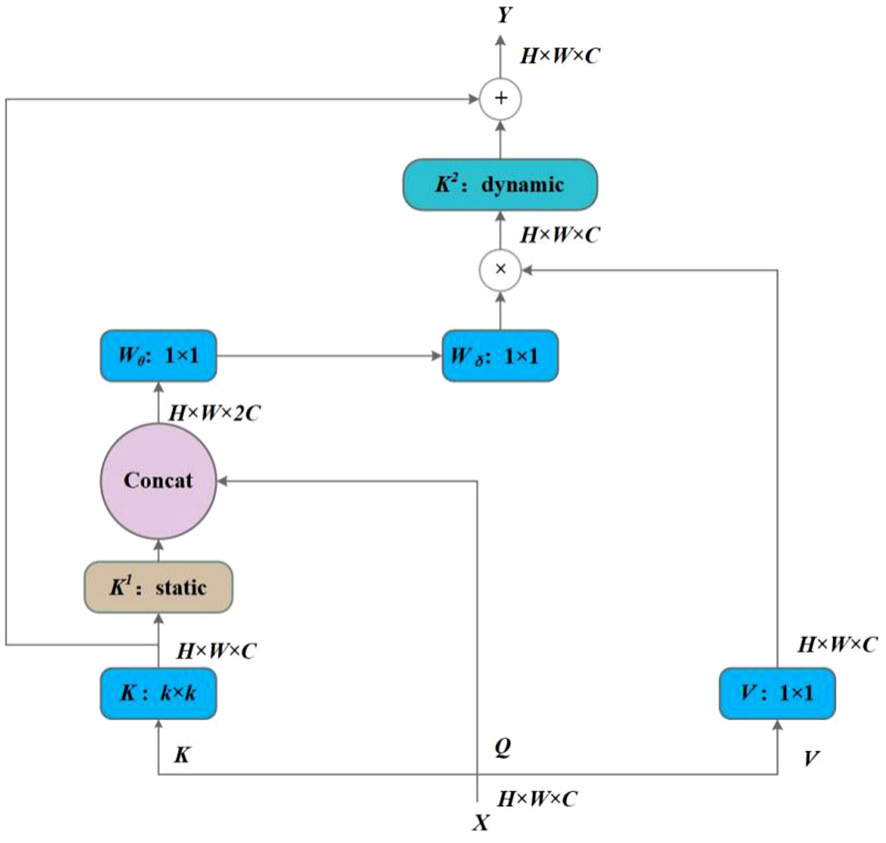
Contextual transformer networks.


The CoT framework is illustrated in **Figure 8**. The input data *X* is of size *H* × *W* × *C*, where *H*, *W*, and *C* are height, width, and number of channels, respectively. The keys, queries, and values are defined as follows Equation 5:

(5) $$\begin{array}{c} K=X,Q=X,V=XW_{\nu } \end{array}$$

*k × k* group convolution is first performed on all neighbor keys to extract the static contextual information, and *K* ^1^ is denoted as the static context representation of input *X*. Then, *K* ^1^ and *Q* are concatenated, and two consecutive 1 × 1 convolutional operations, namely, the *W_θ_* with a ReLU activation function and $W_{\delta }$ without an activation function, are performed subsequently:


(6) $$\begin{array}{c} A=\left[K^{1},Q\right]W_{\theta }W_{\delta } \end{array}$$

From Equation 6, *A* is learned based on the query feature *Q* and the contextualized key feature *K* ^1^, thereby enhancing self-attention learning under the additional guidance of the mined static context *K* ^1^. After that, feature map *K* ^2^ captures the dynamic feature interactions among inputs by aggregating all values *V* and multiplying *A*, where *K* ^2^ is considered a dynamic context. The final output *Y* is the fusion of the static context *K* ^1^ and dynamic context *K* ^2^.

#### 2.2.6 WIoU loss

The Complete Intersection over Union (CIoU) loss function is the default bounding box regression loss function in YOLOv7 and considers differences between the ground truth and predicted bounding boxes in terms of overlap area, center distance, and aspect ratio. However, CIoU exhibits several drawbacks. First, it does not focus on the balance between targets of different scales. Second, in case the aspect ratios of the ground truth and predicted boxes are the same but their width and height values are of great difference, the penalty term fails to accurately reflect the disparity between these two boxes. Third, CIoU increases computational consumption because of the adopted inverse trigonometric function. Therefore, in this study, the CIoU function is replaced with the WIoU loss function, which introduces a dynamic non-monotonic focusing mechanism. The WIoU loss function, proposed by Tong et al. (2023), has developed into three versions: the WIoU_v1_, WIoU_v2_, and WIoU_v3_.

In the WIoU_v1_ algorithm, distance attention is constructed based on the distance metric, obtaining a two-layer attention mechanism. WIoU_v1_ follows Equations 7–9:


(7) $$L_{IoU}=1-IoU=1-\frac{W_{i}H_{i}}{wh+w_{gt}h_{gt}-W_{i}H_{i}}\bar{IoU}$$

(8) $$R_{WIoU}=exp\left(\frac{{\left(x-x_{gt}\right)}^{2}+{\left(y-y_{gt}\right)}^{2}}{\left(W_{g}^{2}+H_{g}^{2}\right)}\right)$$

(9) $$L_{WIoUv1}=R_{WIoU}L_{IoU}$$

where $R_{WIoU}\in \left[1,e\right)\;$ significantly amplifies $L_{IoU}$ of the ordinary-quality anchor box. $L_{IoU}\in \left[0,1\right)$ reduces $R_{WIoU}$ of the high-quality anchor box and its focus on the distance between central points when ground truth matches well with predicted boxes.

Based on the WIoU_v1_, WIoU_v2_ is designed by constructing the monotonic focus coefficient $L_{IoU}^{*}$ , which effectively reduces the contribution of simple samples to loss values, enabling the model to focus on low-quality samples and achieve improved classification performance. The formula of WIoUv2 is shown in Equation 10:


(10) $$L_{WIoUv2}={\left(\frac{L^{*}IoU}{\bar{LIoU}}\right)}^{\gamma }L_{WIoUv1}$$

WIoU_v3_ is designed based on the WIoU_v1_ by constructing the non-monotonic focus coefficient β . To make the model focus on the average quality samples and improve the overall performance, WIoU_v3_ adopts a reasonable gradient gain allocation strategy to dynamically optimize the weight of high- and low-quality anchor boxes in the loss. The formula of WIoUv3 is shown in Equations 11 and 12:


(11) $$\beta =\frac{L_{IoU}^{*}}{\bar{L_{IoU}}}\in \left[0,+\infty \right)$$

(12) $$L_{WIoUv3}=rL_{WIoUv1},r=\frac{\beta }{\delta \alpha^{\beta -\delta }}$$

Compared with WIoU_v1_ and WIoU_v2_, WIoU_v3_ achieved significant improvement on the MS-COCO dataset. In this study, the WIoU_v3_ was employed and replaced the CIoU in YOLOv7.

### 2.3 Performance evaluation

The output of the CSCW-YOLOv7 is a list of detection boxes that contain all weeds with recognition of weed categories. To comprehensively evaluate the performance of the CSCW-YOLOv7, evaluation metrics including precision (*P*), recall (*R*), mean average precision (*mAP*), parameters, and floating-point operations per second (FLOPs) were adopted in this paper. The evaluation metrics are defined as follows:


(13) $$P=\frac{TP}{TP+FP}\times 100\%$$

(14) $$R=\frac{TP}{TP+FN}\times 100\%$$

(15) $$AP=\int_{0}^{1}P\left(R\right)dR$$

(16) $$mAP=\frac{\sum_{i=1}^{n}AP\left(i\right)}{n}$$

In Equation 13, *P* is calculated for a particular weed class by dividing true positives by all positive predictions. In Equation 14, the Recall of a weed class is determined by dividing true positives by the sum of true positives and false negatives. *P* and *R* represent the accuracy of the trained model. In Equation 15, *AP* refers to the area under the curve of *P*–*R* with values ranging from 0 to 1. The higher the *AP*, the better the performance of the deep learning network. In Equation 16, *mAP* is the average of *AP*, where *i* represents a weed category, *AP*(*i*) is the *AP* value of the *i*th weed category, and *n* represents the number of weed categories. Parameters, the number of model parameters, indicate model size and complexity. With a larger number of parameters, models generally require more memory consumption, computation cost, and inference times. FLOPs are used to measure the amount of computation cost of the model, indicating algorithm complexity.

### 2.4 Experimental environment and parameter settings

The processing platform used in this paper is DELL’s Precision T5820X tower graphics deep learning workstation, where the hardware system environment is equipped with Intel i9–10920X CPU (3.5 GHz), NVIDIA GeForce RTX 2080Ti GPU (11 GB of video memory), and 64 GB of RAM. The running environment is Windows 10, in combination with pytorch1.8.1, python3.7, tensorflow-gpu2.3.0, cuda10.2, and cudnn7.6.5 for deep learning model training and testing. The parameters of deep learning models conducted in this experiment are shown in **Table 2**.

**Table 2:** Experimental parameters.

Parameters	Values
Learn rate	0.01
Epochs	200
Batch size	16
Workers	8
Image size	640 × 640

## 3 Results and analysis

### 3.1 Ablation experiments

To evaluate the performance of different modules of the proposed CSCW-YOLOv7 network, an ablation study was conducted, and the results are shown in **Table 3**. The compared components included the CARAFE operator introduced in the up-sampling method, the SE attention network added in the ELAN module, CoT replacing the ELAN module in Neck, and the WIoU loss function. C-YOLOv7, with the adoption of the content-aware CARFE up-sampling operator, achieved 95.5%, 97%, and 94.3% in precision, recall, and *mAP* values, respectively, with a 2% increase in *mAP* values compared with the baseline YOLOv7. In addition, C-YOLOv7 resulted in a 0.4 M and 0.3 G increase in parameters and FLOPs, respectively. This enhancement can be attributed to CARAFE deployment of adaptive up-sampling kernels for diverse feature layers, which accentuates global information and thus improves the *mAP* value. Nevertheless, employing adaptive kernels yielded a slight rise in the number of parameters and FLOPs. Afterward, the SE attention network was added to the ELAN module in the backbone network. The corresponding CS-YOLOv7 achieved 97.5%, 98%, and 94.4% in precision, recall, and *mAP* values, respectively. Compared with the C-YOLOv7, CS-YOLOv7 improved precision, recall, and *mAP* values by 2%, 1%, and 0.1%, respectively, but obtained an increase of only 0.1 M and 0.2 G in parameters and FLOPs, respectively. The results show that the fusion of SE modules into the ELAN network with the integration of image channel features significantly improves the accuracy and robustness of model detection. Furthermore, the CoT network replaced the ELAN network in the Neck of YOLOv7, forming the CSC-YOLOv7. Compared with the CS-YOLOv7, though the precision and *mAP* values of CSC-YOLOv7 slightly decreased by 0.1%, and 0.3%, respectively, the parameters of CSC-YOLOv7 were reduced by 12%, equating to a 4.3-M parameter reduction, which resulted in a 10.4% decrease in FLOPs. The results indicate that the dynamic context mining and self-attention learning mechanisms of the CoT network are efficient with favorable parameter budge. Finally, the CIoU was substituted with WIoU in CSC-YOLOv7, and the corresponding CSCW-YOLOv7 achieved the best performance among the other models. The accuracy, recall, and *mAP* values of the CSCW-YOLOv7 were 97.7%, 98%, and 94.4%, respectively. Compared with those of CSC-YOLOv7, the accuracy and *mAP* were improved by 0.3% and 0.3%, respectively, while the parameters and FLOPs remained the same. These results suggest that the fusion of SE modules into the ELAN network is critical for improving the model’s precision, while the replacement of ELAN with CoT saves some consumption costs. The ablation experiments verified the modified components’ effectiveness in improving the weed detection performance.

**Table 3:** Results of ablation experiments.

Model	CARAFE	SE	CoT	WIoU	Precision	Recall	*mAP*	Parameters (MB)	Model size (MB)	FLOPs (G)
YOLOv7	–	–	–	–	95.9%	97%	92.3%	35.4	71.3	105.2
C-YOLOv7	√				95.5%	97%	94.3%	35.8	72	105.5
CS-YOLOv7	√	√			97.5%	98%	94.4%	35.9	72.3	105.7
CSC-YOLOv7	√	√	√		97.4%	98%	94.1%	31.6	63.7	94.7
CSCW-YOLOv7	√	√	√	√	97.7%	98%	94.4%	31.6	63.7	94.7

C-YOLOv7 adopting CARAFE module; CS-YOLOv7 adopting CARAFE module and SE attention; CSC-YOLOv7 adopting CARAFE module, SE attention, and CoT; CSCW-YOLOv7 adopting CARAFE module, SE attention, CoT, and WIoU.

CoT, contextual transformer; WIoU, Wise Intersection over Union; FLOPs, floating-point operations per second.

"√" indicates that the component is used. "-" indicates that it is not used.

**Figure 9:**
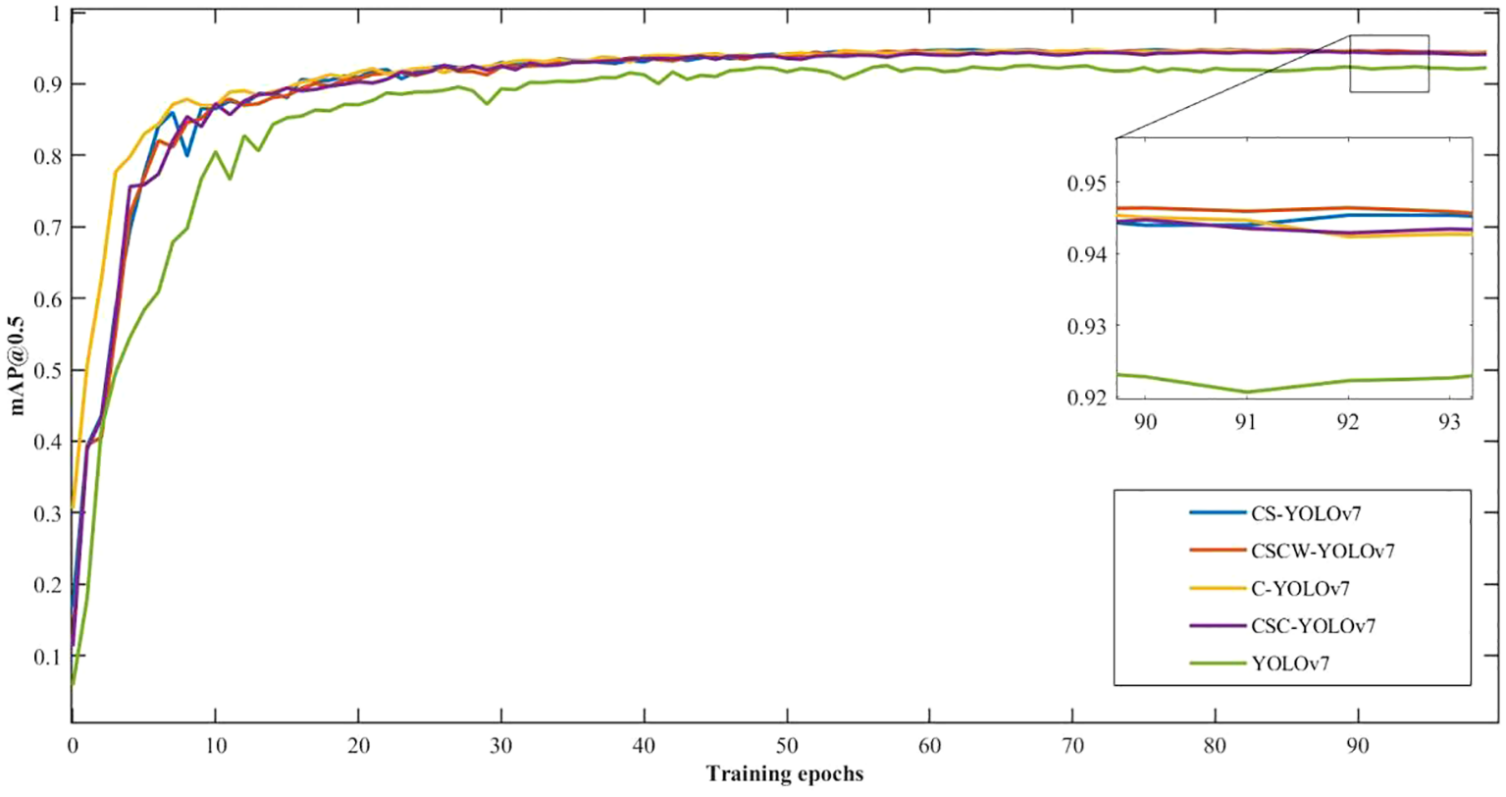
Training curves of *mAP*@0.5 for the improved YOLOv7 models for weed detection.

*mAP*@0.5 is the index of mean average precision with an IoU value of 0.5. **Figure 9** shows the training curves of *mAP*@0.5 for the improved YOLOv7 models in terms of weed detection accuracy. Overall, these models showed promising training performance in terms of fast convergence and high detection accuracies, with more than 92% *mAP*@0.5 attained within 100 training epochs. The training curves showed that the accuracies leveled off beyond 50 epochs for all the models, confirming that training for 100 epochs was sufficient in this study. In **Figure 9**, the improved YOLOv7 models all outperform the YOLOv7. In particular, the proposed CSCW-YOLOv7 and the CS-YOLOv7 showed better performance than the C-YOLOv7 and CSC-YOLOv7. Compared with the CS-YOLOv7, the proposed CSCW-YOLOv7 performed slightly better.

**Figure 10:**
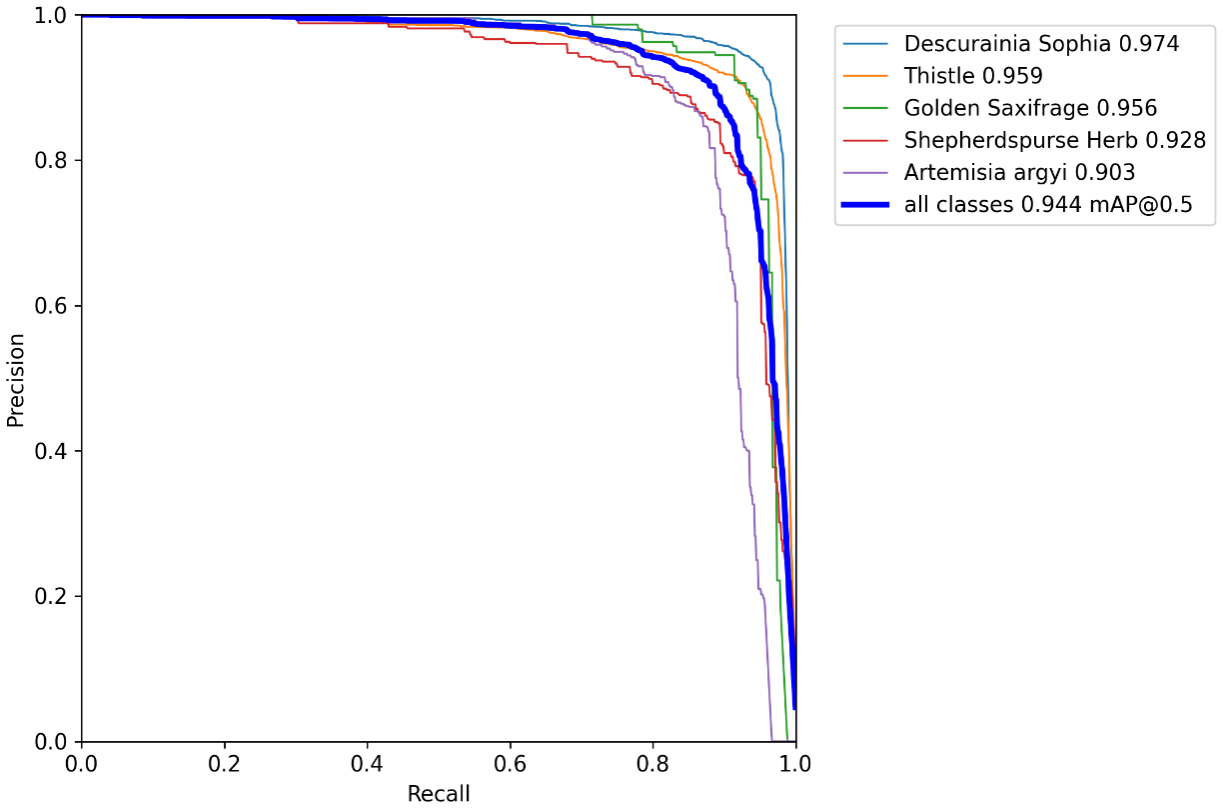
Precision–recall curve of five weed species of the proposed CSCW-YOLOv7 with the horizontal and vertical axes representing recall and precision, respectively.

**Figure 10** displays the precision–recall (PR) curves of five kinds of wheat weeds of the proposed CSCW-YOLOv7 network in the training dataset. Among the five weed species, the closed area composed of the *D. sophia* PR curve is larger than that of the other four species, which indicates that the improved CSCW-YOLOv7 model shows better detection accuracy in *D. sophia*.

**Figure 11:**
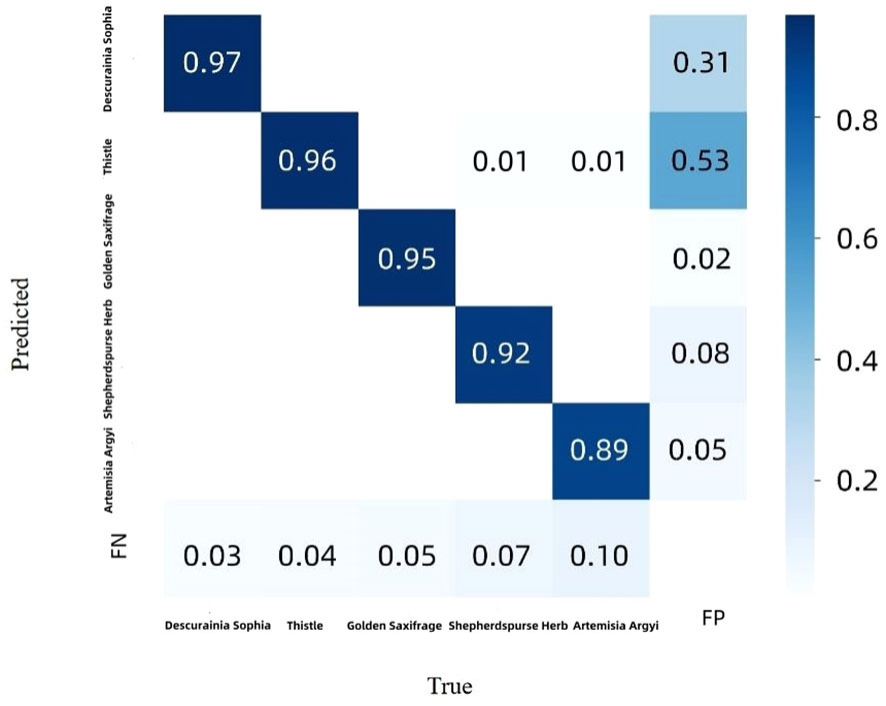
Confusion matrix for CSCW-YOLOv7 evaluated on the test dataset. The rows represent the true labels, while the columns represent the predicted classes.

**Figure 12:**
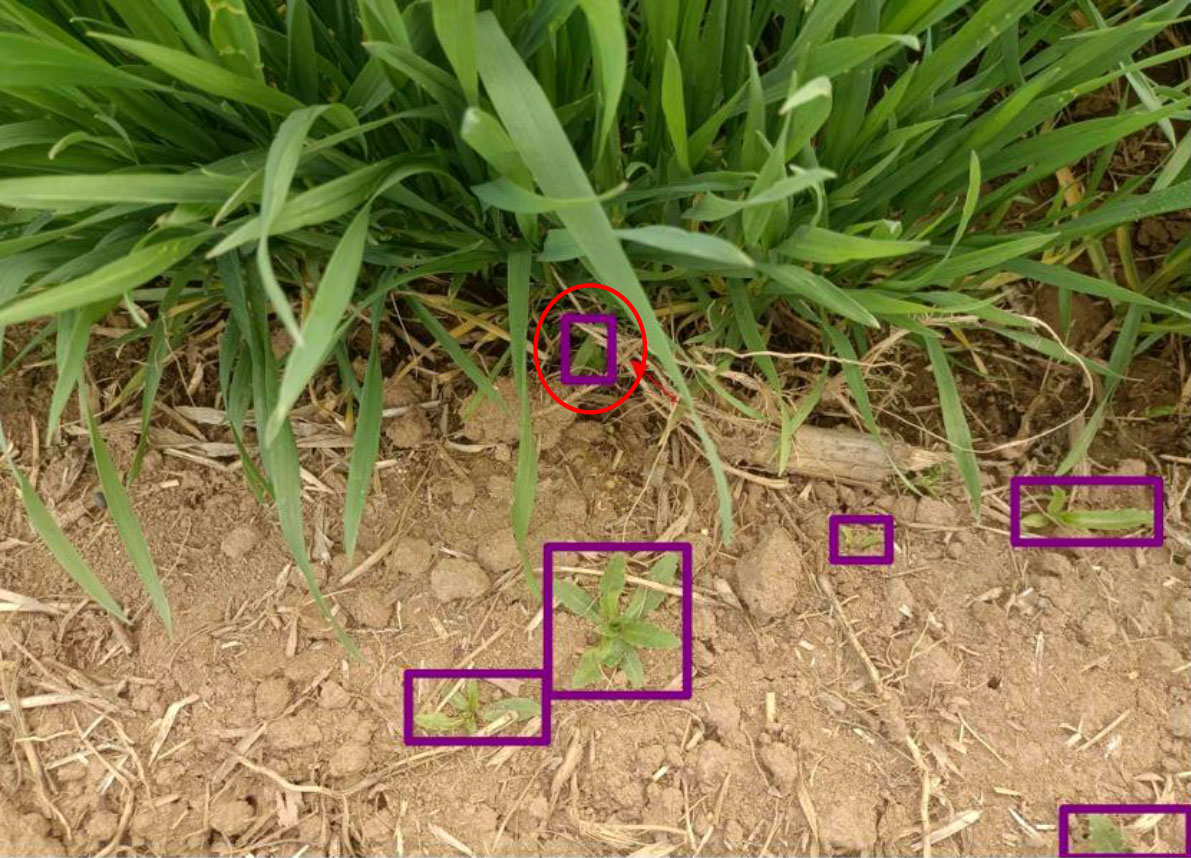
False-positive detection with a wheat leaf incorrectly detected as *Descurainia sophia*. The red colored circle represents false detection.

**Figure 11** shows the confusion matrix of the CSCW-YOLOv7 evaluated on the test datasets. The CSCW-YOLOv7 showed satisfactory results. Accuracies of *D. sophia*, thistle, golden saxifrage, shepherd’s purse herb, and *A. argyi* were 97%, 96%, 95%, 92%, and 89%, respectively, resulting in 93.8% average accuracy. *A. argyi* obtained the lowest accuracy of 89% presumably because *A. argyi* in images is overall comparably small and has a small number of annotations in the training dataset. In particular, *FN* represents false negatives, that is, weeds that were missed detected in our study. *FP* represents false positives, which means that background or wheats were incorrectly detected as one of the five weed species. *FN*s in five weed species account for a small percentage, which was lower than 7%. This result indicated that the CSCW-YOLOv7 has a strong ability to recognize the weed species. However, backgrounds or wheats were likely to be incorrectly detected as weed species. Among the *FP* values, most of the incorrect detections were attributed to *D. sophia* and thistle, with 53% being incorrectly detected as thistle and 31% being incorrectly detected as *D. sophia*. One of the important factors is the similarity in appearance between thistle and wheat. **Figure 12** shows that a wheat leaf was mistakenly detected as a thistle (the red circle) since the wheat leaf is similar to the thistle.

### 3.2 Performance comparison with classical deep learning algorithms

To comprehensively evaluate the effectiveness of the CSCW-YOLOv7 model, the CSCW-YOLOv7 model proposed in this paper was compared with other classical deep learning models, namely, Faster RCNN, YOLOv5m, and YOLOv7. Faster RCNN is a classical two-stage deep learning model, while the YOLOv5m and YOLOv7 are classical one-stage deep learning models. All models were trained and tested on the same dataset, and all the experiments were carried out under the same environment and parameter settings. The comparison results are shown in **Table 4**. The two-stage model, Faster RCNN, obtained average performance, with precision, recall, and *mAP* values of 60.2%, 79.8%, and 79.2%, respectively. However, the one-stage models outperform the Faster RCNN. For instance, YOLOv5m demonstrated precision, recall, and *mAP* of 96.5%, 98%, and 91.3%, respectively, representing an increase of 36.3%, 18.2%, and 12.1% in comparison to Faster R-CNN. In addition, the CSCW-YOLOv7 was superior to other one-stage models. **Table 4** shows that the precision and *mAP* value of the CSCW-YOLOv7 improved by 1.2% and 3.1%, respectively, compared with the YOLOv5m and improved by 1.8% and 2.1% compared with the YOLOv7.

**Table 4:** Performance comparison of CSCW-YOLOv7 and other deep learning models.

Model	Precision	Recall	*mAP*	Parameters (MB)	Model size (MB)	FLOPs (G)
Faster RCNN	60.2%	79.8%	79.2%	136.7	108	401.7
YOLOv5m	96.5%	98%	91.3%	20.8	40.2	48.3
YOLOv7	95.9%	97%	92.3%	35.4	71.3	105.2
CSCW-YOLOv7	97.7%	98%	94.4%	31.6	63.7	94.7

FLOPs, floating-point operations per second.

In the aspect of computational complexity, YOLO models occupied much less memory than the Faster RCNN. Compared with YOLOv5m, YOLOv7 constructs a deeper network structure and adopts a new training strategy; thus, YOLOv7 achieved better detection results but consumed more computation resources and time needed for training. YOLOv7 occupied 71.3 MB of memory, while the YOLOv5m occupied 40.2 MB of memory. The model size of YOLOv5m had compressed by 44% with a 31.1-MB reduction, compared with YOLOv7. Meanwhile, improvement was made on the baseline of the YOLOv7; the improved CSCW-YOLOv7 resulted in a certain degree of compression in model size and improvement in efficiency.

**Figure 13:**
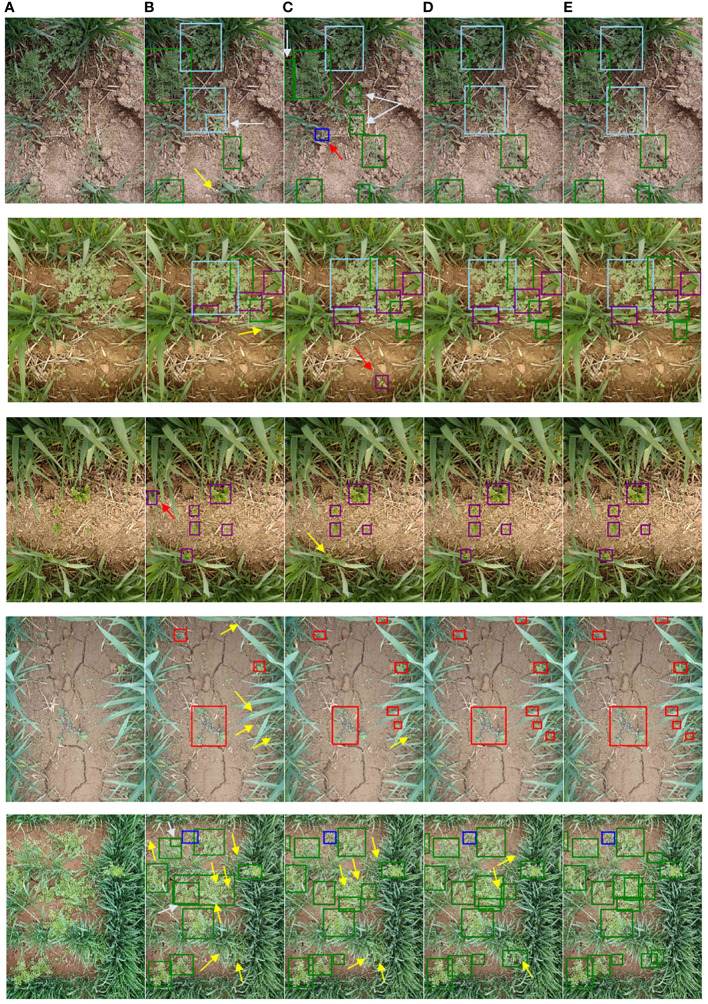
Comparison weed detection results of different deep learning models: **(A)** original image, **(B)** Faster RCNN, **(C)** YOLOv5m, **(D)** YOLOv7, and **(E)** CSCW-YOLOv7. Colors of boxes represent different weed species. Yellow, red, and white arrows point out missed detection, false detection, and repeated detection, respectively.

Accordingly, **Figure 13** shows part of the weed detection performance of four deep learning models, namely, Faster RCNN, YOLOv5m, YOLOv7, and CSCW-YOLOV7. In the first weed image, YOLOv7 and CSCW-YOLOV7 can precisely detect *A. argyi* in the middle of the image. However, Faster RCNN and YOLOv5m detected part of *A. argyi*, resulting in repeated detection. In addition, Faster RCNN missed the detection of *D. sophia*, which was overlapped by wheat, and YOLOv5m falsely recognized an unknown weed as thistle. In the second image, Faster RCNN also missed the detection of *D. sophia*, which was overlapped by wheat, and YOLOv5m yielded a false detection of an unknown weed as thistle. In the third image, Faster RCNN incorrectly detected a leaf of wheat as a thistle, causing a false detection. YOLOv5m missed the detection of a thistle that was occluded by wheat. In the fourth image, several small patches of golden saxifrage were scattered in the wheat field, partially obscured by wheat leaves. YOLOv7 and CSCW-YOLOV7 can precisely detect all the golden saxifrage patches. However, Faster RCNN and YOLOv5m failed to correctly detect all the golden saxifrage patches, and some of them were undetected. The first four images demonstrate that the YOLOv7 and CSCW-YOLOV7 show satisfactory ability in the detection of small-scale and occluded weeds. The fifth image shows a complex wheat field environment; *D. sophia* was densely scattered in the wheat field, and some patches were partially obscured by wheat leaves. Compared with the YOLOv7, the CSCW-YOLOV7 succeeded in recognizing the four densely grown *D. sophia* (in the middle of the image), but the YOLOv7 only detected three of them and failed to detect one.

In summary, occlusion and small scale of weeds are two main factors that affect the recognition of weed species, especially when the detection is performed in a complex field environment. In the case of weeds densely growing in the field, the deep learning models are prone to misclassify the agglomerated entity as one plant (weed); thus, it is easy for weeds to be undetected. In the case of background (leaves of wheat) occlusion, repeated and missed detection are prone to occur. **Table 3** and **Figure 13** demonstrate that the CSCW-YOLOV7 is more sensitive to the weed species and achieves more excellent detection performance in both cases mentioned above.

## 4 Discussion

### 4.1 Analysis of the improvement of YOLOv7

**Figure 14:**
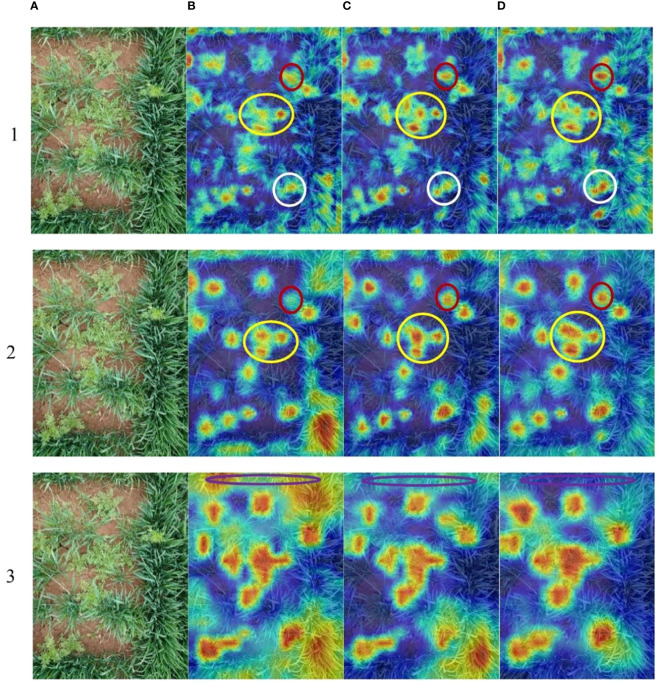
**(A)** Original image. The Grad-CAM of different layers (1-3) of **(B)** YOLOv7, **(C)** CS-YOLOv7,and **(D)** CSC-YOLOv7. The yellow circle depicts that the feature network of improved YOLOv7 can discriminate four densely growing *D. sophia* by four bright areas in the Grad-CAM. In addition, the red and white circles show that the feature network of improved YOLOv7 can recognize the weed that is obscured by wheat. The purple circles indicate that the improved YOLOv7 has advantages in suppressing background features.

In the study, the weed species dataset constitutes a complicated scene with occlusion, overlapping, and weeds of different scales. The results of the ablation studies show the effectiveness of the improved architecture of the CSCW-YOLOV7, including the CARAFE operator introduced in the up-sampling method, SE attention network added in the ELAN module, ELAN module substitution with CoT in Neck, and the WIoU loss function. In **Table 3**, results suggest that improvement in the backbone network and neck is critical for improving the model’s accuracy. To further investigate the impact of different strategies on weed feature extraction, the Gradient-weighted Class Activation Mapping (Grad-CAM) method was adopted. This visualization method can visually display the regions that the backbone network focuses on during the classification process (Selvaraju et al., 2020) by generating a heatmap, helping to gain a deeper understanding of the network’s decision-making process and better explain the mechanisms of the model. In the heatmap generated by the Grad-CAM, the value of each pixel represents its importance to the final target decision. The thermodynamic features of different colors revealed the “attractiveness” of the regional network. Warm colors represent an important impact on the target decision, while cold colors represent a relatively small impact on the target decision. **Figure 14** depicts the Grad-CAM visualizations for different layers of YOLOv7, CS-YOLOv7, and CSC-YOLOv7, presented separately. It is difficult to distinguish two or more weeds when they densely scatter in the field. The yellow circle depicts that the feature network of improved YOLOv7 can discriminate four densely growing *D. sophia* by four bright areas in the Grad-CAM. In addition, the red and white circles show that the feature network of improved YOLOv7 can recognize the weed that is obscured by wheat. The purple circles indicate that the improved YOLOv7 has advantages in suppressing background features. The Grad-CAM shows that the improved YOLOv7 has a better ability of feature extraction and thus can successfully distinguish the obscured and small-scale weeds.

### 4.2 Result comparison with existing solutions

Weed detection based on deep learning goes beyond traditional machine learning techniques that rely on manual design and extraction of features. **Table 5** summarizes the applications of deep learning in weed detection in wheat fields in recent years. Xu et al. (2024) proposed a dual-path weed detection network based on multi-modal information with a weed detection accuracy of 62.3% in a natural wheat field. Haq et al. (2023) implemented deep learning models for weed detection using different frameworks with accuracies of 0.89 and 0.91 for wheat crop weeding. Pérez-Porras et al. (2023) compared six YOLO (v3–v5) DL object-detection models trained on proximal RGB images; YOLOv5s was the top-performing model with test scores of 75.3% F1 scores, 76.2% mean average precision, and 77% accuracy. Saqib et al. (2023) manipulated the dataset by performing image transformation techniques and then trained it on four YOLO models, which showed a *mAP* value of 73.1%. Mishra et al. (2024) proposed a deep learning segmentation model named “Pyramid Scene Parsing Network-USegNet” (PSPUSegNet), and by comparing with UNet, SegNet, and USegNet, etc., PSPUSegNet obtained 96.98% accuracy, 97.98% recall, and 98.96% data accuracy in Deep Weed dataset. Compared to the above studies, the proposed CSCW-YOLOV7 shows promising performance, though the five weed datasets constructed in this paper present a complex phenotype scene with similarity between wheat and weeds, multi-scale weeds, and overlapping weeds.

**Table 5:** Deep learning-based weed detection methods for wheat fields.

Modality	Algorithms	Weed species	Scale of dataset	Accuracy	Reference
RGB and depth images	Faster RCNN	6	7,368	62.3%	(Xu et al., 2024)
RGB images	YOLOv3-Tiny, YOLOv4-Tiny, YOLOv5	1	6,000	91% (YOLOv3-Tiny)	(Haq et al., 2023)
RGB images	YOLOv3-YOLOv5	1	6,319	77% (YOLOv5s)	(Pérez-Porras et al., 2023)
RGB images	YOLOv3, YOLOv3-Tiny, YOLOv4, YOLOv4-Tiny	4	1,065	73.1% (YOLOv4)	(Saqib et al., 2023)
RGB images	PSPUSegNet	6	5,090	96.98%	(Mishra et al., 2024)

### 4.3 Limitation and future application

Although the research object of this study is weeds in wheat, the proposed method is applicable not only to wheat weed detection but also to other types of weeds. By constructing a boarder range of weed datasets, which is then fed into the proposed models for training using transfer learning, it can be used for other types of weed detection. In addition, since most of the weeding robots use embedded devices in the field working environment, further research will be conducted on how to save computational resources under limited hardware configuration to achieve real-time accurate recognition and then migrate the detection model to the embedded device for practical in-field application.

## 5 Conclusion

Weeds are threatening wheat yield by competition with crops for water, light, and nutrients. It is important to adopt weed management to reduce yield losses. However, the similar color, shape, and occlusion between wheat and weeds pose a challenge to the detection of weeds in wheat fields. Therefore, the precise detection of weeds in the field is the premise of implementing weed management. The conclusions are as follows:

1) A dataset was constructed for five weeds that are commonly found in wheat fields, namely, *D. sophia*, thistle, golden saxifrage, shepherd’s purse herb, and *A. argyi*. A CSCW-YOLOv7 based on improved YOLOv7 architecture was constructed to detect and recognize the weeds under the complex field environment. In the CSCW-YOLOv7, the CARAFE operator was introduced into the YOLOv7 network as an up-sampling algorithm to improve the recognition of small targets. Then, the SE network was added to the ELAN module in the backbone network and the concatenation layer in the feature fusion module to enhance important weed features and suppress irrelevant features. In addition, the CoT module, a transformer-based architectural design, was used to capture global information and enhance self-attention by mining contextual information between neighboring keys. Finally, the WIoU loss function introducing a dynamic non-monotonic focusing mechanism was employed to better predict the bounding boxes of the occluded weed.

2) To verify the practicability of the CSCW-YOLOv7, model performances were comparatively evaluated and compared with classical deep learning models. The ablation experiment results showed that the proposed CSCW-YOLOv7 achieved the best performance among the other models. The precision, recall, and *mAP* values of the CSCW-YOLOv7 were 97.7%, 98%, and 94.4%, respectively, which were 1.8%, 1%, and 2.1% better than the baseline YOLOv7. Meanwhile, the parameters were compressed by 10.7% with a 3.8-MB reduction, resulting in a 10% decrease in FLOPs. The Grad-CAM visualization method suggested that the CSCW-YOLOv7 can learn a more representative set of features that helped better locate the weeds of different scales in complex field environments. In addition, the performance of the CSCW-YOLOv7 was compared to the widely used state-of-the-art deep learning models, and results indicated that the CSCW-YOLOv7 exhibits better ability to distinguish the overlapped weeds and small-scale weeds. The overall results suggest that the CSCW-YOLOv7 is a promising tool for the detection of weeds in wheat fields and has great potential for field applications.

## Data availability statement

The raw data supporting the conclusions of this article will be made available by the authors, without undue reservation.

## Author contributions

KW: Conceptualization, Data curation, Formal analysis, Investigation, Methodology, Software, Validation, Writing – original draft, Writing – review & editing. ML: Conceptualization, Software, Writing – original draft. HZ: Data curation, Methodology, Software, Validation, Writing – original draft. XH: Data curation, Validation, Writing – review & editing, Conceptualization. CL: Software, Writing – review & editing, Data curation. YL: Data curation, Writing – review & editing. LZ: Data curation, Writing – review & editing, Resources. HL: Conceptualization, Resources, Supervision, Writing – review & editing, Methodology, Project administration, Visualization. ST: Conceptualization, Data curation, Formal analysis, Funding acquisition, Investigation, Methodology, Project administration, Resources, Software, Supervision, Validation, Visualization, Writing – original draft, Writing – review & editing.
